# Fabrication and characterization of dual-functional ultrafine composite fibers with phase-change energy storage and luminescence properties

**DOI:** 10.1038/srep40390

**Published:** 2017-01-09

**Authors:** Peng Xi, Tianxiang Zhao, Lei Xia, Dengkun Shu, Menjiao Ma, Bowen Cheng

**Affiliations:** 1 Tianjin Polytechnic University, 300387 Tianjin, P.R. of China; 2 State Key Laboratory of Separation Membranes and Membrane Processes, Tianjin 300387, P.R. of China

## Abstract

Ultrafine composite fibers consisting of a thermoplastic polyurethane solid-solid phase-change material and organic lanthanide luminescent materials were prepared through a parallel electrospinning technique as an innovative type of ultrafine, dual-functional fibers containing phase-change and luminescent properties. The morphology and structure, thermal energy storage, and luminescent properties of parallel electrospun ultrafine fibers were investigated. Scanning electron microscopy (SEM) images showed that the parallel electrospun ultrafine fibers possessed the desired morphologies with smaller average fiber diameters than those of traditional mixed electrospun ultrafine fibers. Transmission electron microscopy (TEM) images revealed that the parallel electrospun ultrafine fibers were composed of two parts. Polymeric phase-change materials, which can be directly produced and spun, were used to provide temperature stability, while a mixture of polymethyl methacrylate and an organic lanthanide complex acted as the luminescent unit. Differential scanning calorimetry (DSC) and luminescence measurements indicated that the unique structure of the parallel electrospun ultrafine fibers provides the products with good thermal energy storage and luminescence properties. The fluorescence intensity and the phase-change enthalpy values of the ultrafine fibers prepared by parallel electrospinning were respectively 1.6 and 2.1 times those of ultrafine fibers prepared by mixed electrospinning.

In the energy industry, issues of efficiency and environmental impact have become causes of serious global concern in recent years^1^,^2^,^3^. Pollution-heavy industries, such as traditional printing and dyeing, have sought to update their existing technology to mitigate such problems. Intelligent chromic and luminescent fibers can change color in response to various external stimuli and are therefore an attractive alternative to dyed and chromic fabrics^4^,^5^,^6^. However, such chromic and luminescent fabrics have two key limitations. The thermal stability of chromic and luminescent materials is poor, making such fibers difficult to fabricate through conventional melt-spinning methods. Therefore, new methods have been developed to improve the thermal stability of these materials. For example, organic luminescent lanthanide complexes have been incorporated into mesoporous and macroporous materials by wet impregnation, ion exchange methods and covalent graft technology^7^,^8^,^9^. However, similar to inorganic luminescent materials, the compatibility of these materials with polymers is poor; after a period of time, the organic lanthanide complexes inevitably precipitate. The other main limitation is that the fluorescence intensity of luminescence materials is temperature dependent^10^,^11^,^12^, which can directly affect the color purity of the fabric.

Phase-change materials (PCMs) are important functional materials that can be a constant heat source at the phase-change temperature and can maintain a constant temperature^13^. Furthermore, their phase-changes are reversible, which allows PCMs to be used repeatedly. Based on the phase-change properties of PCMs, some phase-change fibers have been designed and produced through blending^14^, graft polymerization^15^, and copolymerization^16^. Electrospinning is a simple, convenient, and versatile technique for generating ultrafine fibers at room temperature or lower temperatures. Many types of ultrafine fiber have been fabricated with this method, and they have been used in healthcare^17^, biotechnology^18^, environmental engineering^19^, and energy storage^20^. It is worth noting that large-scale production of ultrafine fibers has also been realized through electrospinning. If we can combine the intelligent controlled-temperature property of PCMs with electrospinning at low temperatures, high-quality chromic and luminescent fabrics could be successfully prepared.

With this in mind, we selected various PCMs (polyethylene glycol (PEG), stearic acid, and paraffin) and mixed them with organic luminescent materials. Subsequently, electrostatic spinning was used to prepare ultrafine fibers from these mixtures. However, the initial results of combining luminescent materials with these PCMs indicated that the crystallization temperatures of the PCMs were reduced, and the degree of supercooling in the PCM increased. This indicates that the PCMs lost their controlled-temperature property after this process. Furthermore, when low-molecular-weight PCMs were added to fiber-forming polymers, many convex and concave defects were present on the fiber surfaces, which can seriously affect the mechanical properties of the fibers and fabrics. Improving the electrospinning method and selecting suitable PCMs are therefore new challenges for successfully preparing chromic and luminescent fibers and fabrics from these materials.

In this research, dual-functional ultrafine composite fibers with phase-change energy storage and luminescence properties were successfully prepared using a parallel electrospinning technique. In this dual-functional ultrafine fiber, a thermoplastic polyurethane solid-solid phase-change material (PUPCM)^21^, which can be directly produced and spun, was used to provide temperature stability, while a mixture of polymethyl methacrylate (PMMA) and an organic lanthanide complex (TbL) acted as the luminescent component. The as-prepared ultrafine fiber samples have good fluorescence properties and emit a bright green light due to the introduction of the polymeric PCM. The fluorescence intensity of these new dual-functional ultrafine fibers is more than 2.5 times that of PMMA ultrafine luminescent fibers, and the as-prepared dual-functional ultrafine fibers have better luminescence properties in the temperature range of 20–80 °C. Furthermore, the phase-change enthalpy of the PCMs obviously improved, and the phase-change temperature range also narrowed from 18–55 °C to 25–48 °C due to the nano-size effects of the as-prepared dual-functional ultrafine fibers.

## Results

### Influence of PMMA on the PMMA/PUPCM ultrafine composite fibers morphology

**Figure 1:**
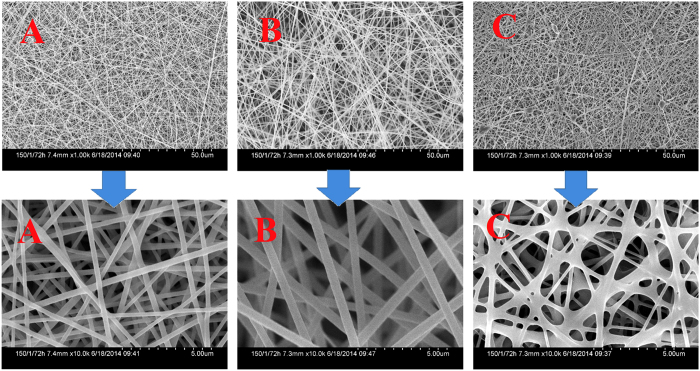
SEM images of PMMA/PUPCM ultrafine composite fibers. The mass ratios of PMMA and PUPCM were (**A**) 70:30, (**B**) 50:50, and (**C**) 30:70.

Due to the coordination between lanthanide ions and ester groups in PMMA molecules, high concentrations of organic rare earth complexes can be evenly dispersed into PMMA; we therefore selected PMMA as one component of our dual-functional ultrafine fibers. Figure 1 shows scanning electron microscopy (SEM) images of the PMMA/PUPCM ultrafine composite fibers. Compared with other organic PCMs, when the mass percent of PUPCM is less than 70%, the resulting PMMA/PUPCM ultrafine composite fibers have smooth, uniform surfaces. This verifies that PUPCM has good fiber-forming performance. When the mass percent of PUPCM reaches 70%, the as-prepared PMMA/PUPCM ultrafine composite fibers show a tubercle fiber structure (Fig. 1C). This was attributed to the aggregation of the excessive phase-change materials during electrospinning process^22^. Furthermore, PMMA is beneficial for improving the apparent morphologies of the as-prepared fibers. As the mass percent of PMMA increases, the fiber surfaces become smoother, and the average fiber diameter decreases. Thus, we selected a 1:1 mass ratio of PMMA and PUPCM during the electrospinning process for dual-functional ultrafine fibers.

### Morphology and properties of mixed electrospun dual-functional ultrafine fibers

**Figure 2:**
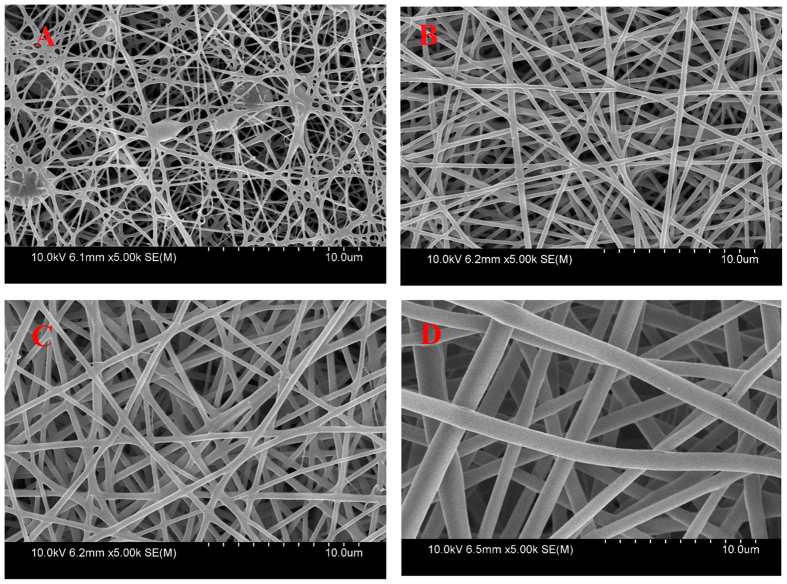
SEM images of mixed electrospun ultrafine fibers at polymer concentrations of 20% (**A**), 24% (**B**), 28% (**C**), and 32% (**D**).

**Figure 3:**
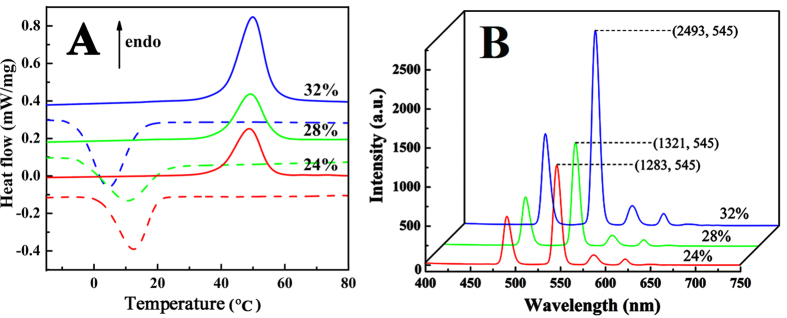
DSC curves (**A**) and fluorescence emission spectra (**B**) of mixed electrospun ultrafine fibers with polymer concentrations of 24%, 28%, and 32%.

Figure 2 shows SEM images of mixed electrospun ultrafine fibers. At a polymer concentration of 24%, the as-prepared mixed electrospun fibers show better morphologies. The diameters of the as-prepared mixed electrospun fibers are 300–1500 nm. Figure 3A shows differential scanning calorimetry (DSC) curves of the mixed electrospun ultrafine fibers; these data are summarized in Table 1. The results indicate that the mixed electrospun fibers with 24% and 28% solid contents have inferior phase-change performances. The phase-transition enthalpy values of these mixed electrospun fibers are only 20% of those values for the corresponding pure PUPCM. Although the phase-transition enthalpy was increased to a certain extent in the mixed electrospun fibers with 32% solid content, the average diameter of the as-prepared fiber samples is approximately 1.5 μm, which limits their widespread applications in many fields. The main reason for the low phase-change functions in the mixed electrospun fibers is that the amorphous polymer, which was mixed and dispersed in the molecular structure of the PCMs, hinders the crystallization, reduces the phase-transition enthalpy values, and affects the temperature stability of the mixed electrospun ultrafine fibers.

Figure 3B presents the fluorescence emission spectra of the mixed electrospun ultrafine fibers. With the increase in the phase-change functions of the as-prepared mixed electrospun ultrafine fibers, the fluorescence intensity of the as-prepared fibers clearly improves. These results verify that PUPCM can improve the transition environment of rare earth ions and sensitize the rare earth ions for fluorescence. However, the phase-transition enthalpy values of the mixed electrospun ultrafine fibers are too low to satisfy the requirement of better temperature stability behavior.

**Figure 4:**
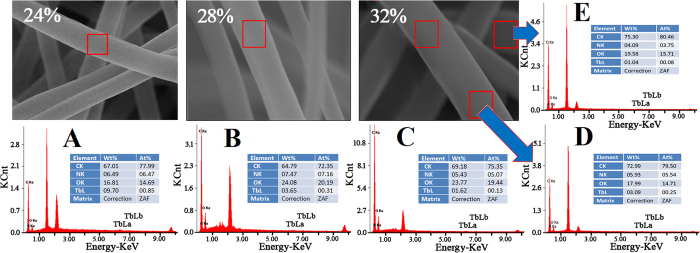
EDS spectra of mixed electrospun ultrafine fibers with polymer concentrations of 24% (**A**), 28% (**B**), and 32% (**C**,**D** and **E**).

Figure 4 gives energy dispersive spectrometer (EDS) spectra of the mixed electrospun ultrafine fibers. As shown in Fig. 4, the percentage contents of TbL for 24%, 28%, and 32% solid-content mixed electrospun ultrafine fibers are 0.85, 0.31, and 0.13, respectively. Even in different locations of the same fiber, the contents of TbL are also different (Fig. 4D). The results indicate that TbL cannot be evenly dispersed in the mixed electrospun ultrafine fibers, affecting the luminescent properties of the mixed electrospun ultrafine fibers.

### Morphology and structure of parallel electrospun dual-functional ultrafine fibers

**Figure 5:**
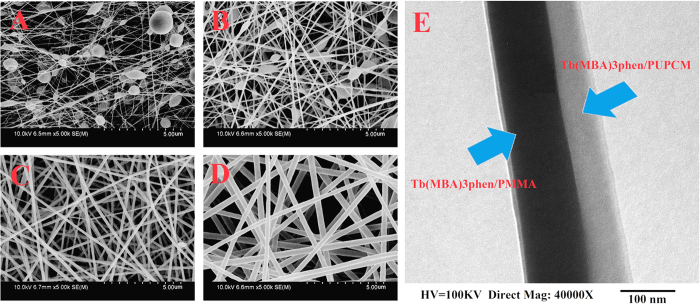
SEM images of parallel electrospun ultrafine fibers with polymer concentrations of 16% (**A**), 20% (**B**), 24% (**C**), and 28% (**D**); TEM image of fiber (**E**).

Parallel electrospinning was used to prepare dual-functional ultrafine fibers. SEM images of the as-prepared parallel electrospun ultrafine fibers are shown in Fig. 5A–D. Similar to the mixed electrospun fibers, when the polymer concentration was 24% or higher, the parallel electrospun ultrafine fibers have good morphologies. The diameters of the as-prepared fibers were 100–250 nm, and the fiber surfaces were smooth. Figure 5E shows TEM images of the parallel electrospun ultrafine fibers. Figure 5E indicates that the as-prepared fibers are composed of two parts; the light regions are PUPCM, and the dark parts are PMMA and TbL particles. As shown by the SEM and TEM analysis, parallel electrospun ultrafine fibers were successfully prepared.

### Phase-change properties of parallel electrospun ultrafine fibers

**Figure 6:**
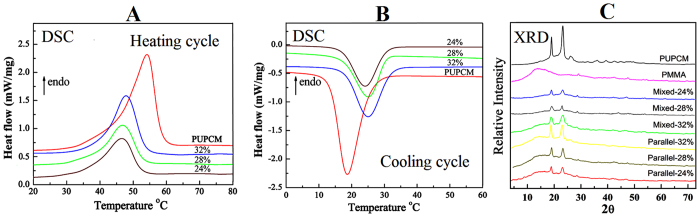
DSC (**A**,**B**) and XRD (**C**) curves of parallel electrospun ultrafine fibers and PUPCM.

The DSC and X-ray diffraction (XRD) curves were collected and used to evaluate the phase-change properties of the parallel electrospun ultrafine fibers (Fig. 6). The DSC analysis indicates that the phase-transition enthalpy value of the parallel electrospun ultrafine fiber was greater than that of the mixed electrospun fiber at the same polymer concentration (Table 1). At polymer solid contents of 24%, 28% and 32%, the phase-change enthalpies of the as-prepared parallel electrospun ultrafine fibers were 47.15, 59.21, and 80.99 J/g, respectively. In Fig. 6C, the parallel electrospun ultrafine fibers, mixed electrospun ultrafine fibers and pure PUPCM show similar XRD curves; this verifies that these samples have similar phase-change units^23^. The detail crystalline parameters of mixed and parallel electrospun ultrafine fibers were shown in Table S1. Compared to the mixed spun ultrafine fibers, the parallel spun ultrafine fibers have higher crystallinity, giving them better phase-transition enthalpy and temperature stability^24^. Note that the phase-transition temperature range changed to 25–48 °C (from 18–55 °C for PUPCM) in the parallel spun ultrafine fibers. As a result, the supercooling of these PCMs decreased. This is important for improving the controlled-temperature properties of PCMs and for increasing the fluorescence intensity of rare earth complexes.

### Fluorescent properties of parallel electrospun ultrafine fibers

**Figure 7:**
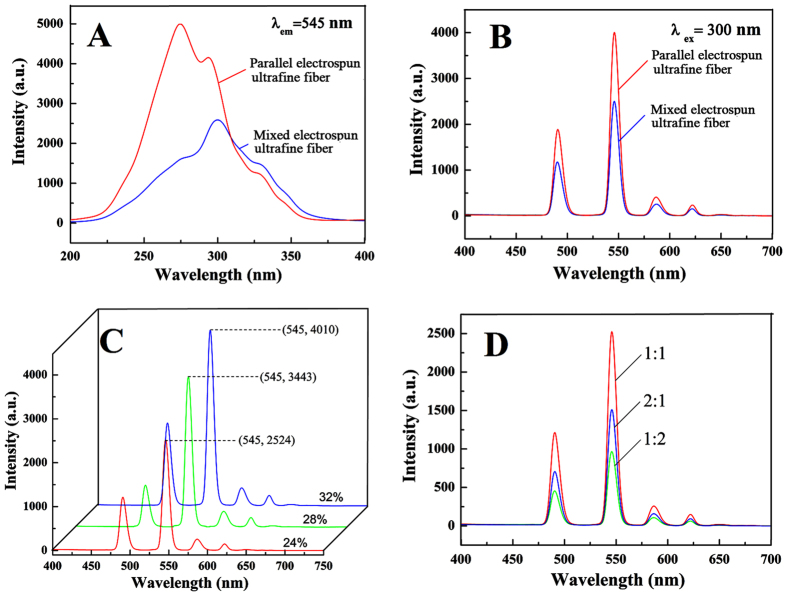
Fluorescence excitation (**A**) and emission spectra (**B**) of the parallel electrospun ultrafine fibers; Emission spectra of parallel electrospun ultrafine fibers containing different solid contents (**C**) and mass ratios of PMMA and PUPCM (**D**).

Figures 7A and B respectively show the fluorescence excitation and emission spectra of the parallel electrospun ultrafine fibers. For comparison, the fluorescence spectra of the mixed electrospun ultrafine fibers are also shown in Fig. 7. The excitation spectrum of the parallel electrospun ultrafine fiber shows an intense broad band at 250–350 nm, with peaks at 275, 294, and 331 nm. The emission spectra of the parallel and mixed electrospun ultrafine fibers are similar. Here, the emission peaks located at 485, 545, 580, and 620 nm belong to ^5^D_4_ → ^7^F_j (j=6,5,4,3)_ characteristic emissions of Tb^3+^ ions^25^. The strongest emission is ^5^D_44_ → ^7^F_5_. The strongest emission peak intensity of the parallel electrospun ultrafine fibers is 1.6 times that of the mixed electrospun ultrafine fibers. This indicates that Tb^3+^ ions can be efficiently sensitized and excited in parallel electrospun ultrafine fibers.

Figure 7C shows the emission spectra of parallel electrospun ultrafine fibers containing different solid contents. As the solid contents increased, the fluorescence intensity of the as-prepared fiber samples gradually increases. At solid contents of 24%, 28%, and 32%, the fluorescence intensities of the as-prepared fiber samples are 2524, 3443, and 4010, respectively. In the mixed electrospun ultrafine fibers, the fluorescence intensities corresponding to the same fiber solid contents are 1283, 1321, and 2493, respectively. This indicates that the ultrafine fibers prepared using parallel electrospinning have higher fluorescence intensities than the fibers fabricated using mixed electrospinning. Figure 7D shows the emission spectra of parallel electrospun ultrafine fibers containing different mass ratios of PMMA and PUPCM. At a PMMA: PUPCM ratio of 1:1, the prepared parallel electrospun ultrafine fibers have the strongest fluorescence intensity compared to the other ratios.

## Discussion

### Crystallinity and phase-change mechanisms of parallel electrospun dual-functional ultrafine fibers

The crystallization performance of parallel electrospun dual-functional ultrafine fibers is one of the important factors to determine their phase-change functions. Figure 6C shows the XRD patterns of pure PUPCM, electrospun PMMA fibers, and parallel electrospun ultrafine fibers. The PMMA fibers have a broad diffraction peak in the range of 10° to 20°, which indicates that the PMMA fibers are amorphous. Meanwhile, the parallel electrospun ultrafine fibers have two strong diffraction peaks at about 2θ = 19.1° and 2θ = 23.2°, which is same as pure PUPCM. Therefore, the diffraction peaks in the spectra of the parallel electrospun ultrafine fibers are produced by PUPCM.

**Figure 8:**
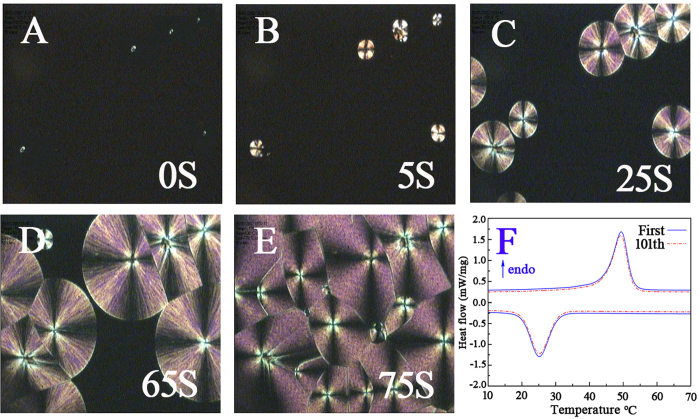
Crystallization processes (**A**–**E**) and DSC curve after 100 thermal cycles (**F**) of parallel electrospun ultrafine fibers with polymer concentrations of 32%.

Theoretically, the phase-change enthalpy values of the electrospun ultrafine fibers can be obtained by multiplying the phase-change enthalpy value of pure PUPCM and the mass percent of PUPCM in the fibers. However, Table 1 shows that the real phase-change enthalpy values of the mixed electrospun ultrafine fibers are obviously lower than the corresponding theoretical values. The main reasons for this difference are that the solvent evaporates in milliseconds during the electrospinning process for the mixed electrospun ultrafine fibers, and the PUPCM molecules may not have enough time to form well-defined crystallites in the composite fibers. Furthermore, the amorphous PMMA molecules are dispersed around the PUPCM molecules, and they limit the movement of PUPCM molecules during the crystallization processes. These results cause a retardation of the crystallization of PUPCM in the composite fibers and further result in the decrease in phase-change enthalpy values^26^. In the structure of the parallel electrospun ultrafine fibers, PUPCM is a relatively independent component. The dilution and crystallization-limiting effects of amorphous PMMA molecules are therefore prevented. Moreover, the TbL located around the PUPCM molecules acts as a nucleating agent during the crystallization processes. The results improve the crystallization properties of parallel electrospun ultrafine fibers and endow the parallel electrospun ultrafine fibers with good phase-change functions. Figure 8A–E displays the crystallization processes of 32% solid content parallel electrospun ultrafine fibers using a polarizing microscope with a heating and freezing stage. The results suggest that parallel electrospun ultrafine fibers require only 65 seconds to complete the spherulite growth process and have good spherulite morphologies. Figure 8F shows the DSC curve of 32% solid content parallel electrospun ultrafine fibers after 100 thermal cycles in the temperature range of 10 °C to 70 °C. Note there is almost no change in the melting or crystallization point of the as-prepared sample after 100 cycles. The results indicated that 32% solid content parallel electrospun ultrafine fibers have very good thermal reliability during their phase-change process.

### Luminescence and temperature stability mechanisms of parallel electrospun dual-functional ultrafine fibers

Figure S2A describes the temperature dependence of the relative emission peak intensity for PMMA luminescent fibers and parallel electrospun ultrafine fibers. The emission intensity of the as-prepared PMMA luminescent fibers decreases to approximately 70.0% at 80 °C. As the sample temperature increases, the fluorescence intensity decreases, and temperature quenching occurs. This is because higher temperatures promote nonradiative relaxation for both cascade multiphonon transitions and energy transfers originating from the luminescent level^27^. However, in parallel electrospun ultrafine fibers, the change in the fluorescence intensity is small compared to the increase in temperature in the range of 20–80 °C. This demonstrates that the fluorescence intensity of parallel electrospun ultrafine fibers can be held constant within a certain range of temperature changes in PUPCM. Figures S2B and C show changes in the fluorescence lifetime and quantum yield of parallel electrospun ultrafine fibers with the increasing temperature. The results verify that changes in fluorescence lifetime and quantum yield of parallel electrospun ultrafine fibers are quite low in the temperature range of 25–80 °C. This stability depends on the excellent phase-change functions of the parallel electrospun ultrafine fibers.

In summary, we prepared dual-functional ultrafine fibers with phase-change energy storage and luminescence properties using parallel electrospinning. As the solid contents of the polymers increased, the luminescence intensities and phase-change enthalpy values of the as-prepared parallel ultrafine fibers were enhanced. With the same solid contents, the fluorescence intensity and phase-change enthalpy values of ultrafine fibers prepared by parallel electrospinning were 1.6 and 2.1 times that of ultrafine fibers prepared by mixed electrospinning, respectively. At the same time, the presence of PUPCMs effectively prevented the fluorescence intensity of as-prepared parallel ultrafine fibers from decreasing with the increase in temperature in the range of 20–80 °C. The results are of great significance for extending the applications of rare earth luminescent fibers into fluorescent displays and fluorescent color clothing.

## Methods

### Materials

TbL, Tb(4-methylbenzoic acid)_3_phen, was prepared as reported previously^28^. PMMA (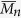 = 31,000 g/mol, 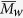
*/*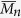 = 1.12) was purchased from Sigma-Aldrich Co. LLC., China. The polymeric phase-change material (PUPCM, 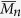 = 36,000 g/mol) was synthesized according to previously published procedures^21^. Other solvents were purchased from Tianjin Keetong Chemical Reagent Co. Inc., China, and dried before use.

### Mixed electrospun dual-functional ultrafine fibers

To determine the best mass ratio of PMMA to PUPCM, ultrafine composite fibers of PMMA/PUPCM were prepared using a traditional single-spinneret electrospinning method. First, 0.31 g of combined PMMA and dried PUPCM were added to freshly distilled DMF at PUPCM/PMMA mass ratios of 30%, 50% and 70%; the total polymer concentration was fixed at 24% with respect to the solvent. The mixture was mixed and stirred at 60 °C until the solution became clear.

To obtain mixed electrospun dual-functional ultrafine fibers, 0.30 g PMMA and 0.30 g PUPCM were first added to freshly distilled DMF and stirred at 50 °C until the solution became transparent. The TbL powder was then introduced to the mixture, and the mass percent of TbL with respect to the forming polymers was 3%. Finally, the mixed solution was stirred at 65 °C for 5 h, and the luminescent material was evenly dispersed in the spinning solution.

The as-prepared spinning solutions were placed in a 10 mL syringe fitted with a metallic needle with an inner diameter of 0.41 mm. The syringe was fixed horizontally on the syringe pump (LSP02-1B, Baoding Longer Precision Pump Co. Ltd., China), and the electrode of the high voltage power supply (Changzhou Blue-Butterfly Automatic Control Fittings Factory, China) was clamped to the metal needle tip. The most uniform ultrafine fibers were obtained with a feed rate of 0.5 mL/h, an applied voltage of 15 kV, and a tip-to-collector distance of 25 cm; these parameters were used for the electrospinning of the above polymer solutions. A grounded stationary rectangular metal collector covered by a piece of aluminum foil was used as the target for ultrafine fibers deposition. The electrospinning was carried out at 26 °C and 32% relative humidity (R.H.) in air. All nonwoven fibrous mats were vacuum dried at room temperature for 3 days to completely remove any solvent residue prior to electrospinning.

### Electrospinning of parallel electrospun dual-functional ultrafine fibers

For the parallel electrospinning solution, 0.30 g PMMA, 0.009 g TbL and 5 mL DMF were mixed and stirred at 60 °C for 2 h to prepare the uniform electrospinning solution 1. For solution 2, 0.30 g PUPCM, 0.009 g TbL and 5 mL DMF were mixed and stirred at 65 °C for 3 h. The electrospinning solutions with different (polymer concentrations were obtained by changing the dosages of DMF.

Spinning solutions 1 and 2 were injected into the two syringes, and the distance between the tip and the spinneret was 20 cm. A direct current voltage of 15 kV was applied between the spinneret and the collector to generate stable and continuous parallel electrospun dual-functional ultrafine fibers. The feed rate was 3 mL/h, the spinning temperature was 22 °C, and the atmospheric R.H. was 47%.

### Analysis methods

Analysis methods were introduced in supporting information.

## Additional Information

**How to cite this article**: Xi, P. *et al*. Fabrication and characterization of dual-functional ultrafine composite fibers with phase-change energy storage and luminescence properties. *Sci. Rep.* **7**, 40390; doi: 10.1038/srep40390 (2017).

**Publisher's note:** Springer Nature remains neutral with regard to jurisdictional claims in published maps and institutional affiliations.

## Supplementary Material

Supporting Information

## Figures and Tables

**Table 1:** The phase-change and luminescent properties of parallel electrospun ultrafine fibers.

Samples	Phase-change properties				Luminescent properties		
	∆H (J/g)		T_p_ (°C)		Intensity (^5^D_4_ → ^7^F_5_)	Luminescent Lifetime (ms)	Quantum Yield (%)
	Heating Cycle	Cooling Cycle	Heating Cycle	Cooling Cycle			
PUPCM	128.15	117.93	55.10	18.72	—	—	—
PMMA nanofiber	—	—	—	—	1576	0.64	32.13
Mixed electrospun ultrafine fiber with 24% solid content	22.11	23.70	49.10	12.41	1283	0.58	28.12
Mixed electrospun ultrafine fiber with 28% solid content	22.38	25.73	48.71	10.40	1321	0.61	28.54
Mixed electrospun ultrafine fiber with 32% solid content	38.56	34.43	50.12	4.41	2493	0.62	31.98
Parallel electrospun ultrafine fiber with 24% solid content	47.15	42.83	47.65	24.82	2524	0.66	32.45
Parallel electrospun ultrafine fiber with 28% solid content	59.21	49.34	47.76	25.02	3443	0.72	35.12
Parallel electrospun ultrafine fiber with 32% solid content	80.99	65.48	48.21	25.13	4010	0.85	35.67

*Tp* = Peak transition temperature of samples; ∆*H* = phase-transition enthalpy of samples. The quantum yield of the samples were calculated according to the reference, as reported previously^29^.
