# Supplementary material for: Fabrication and characterization of dual-functional ultrafine composite fibers with phase-change energy storage and luminescence properties
Source: Sci Rep. 2017 Jan 9;7:40390. doi: 10.1038/srep40390 (PMC5220296; doi:10.1038/srep40390)
Supplement: Supporting Information [file srep40390-s1.pdf]

Fabrication and characterization of dual-functional ultrafine composite fibers with phase-change energy storage and luminescence properties

Peng Xi[1, 2,*], Tianxiang Zhao[1, +], Lei Xia[1, 2 +], Dengkun Shu[1, +], Menjiao Ma[1, +] and Bowen Cheng[1, 2*]

[1]State Key Laboratory of Separation Membranes and Membrane Processes, Tianjin 300387, P.R. of China.

[2]Tianjin Polytechnic University, 300387 Tianjin, P.R. of China

*Corresponding authors: xpsyq0007@sina.com; Bowen@tjpu.edu.cn

[+]These authors contributed equally to this work

Analysis methods. SEM (Hitachi S4800, Japan) and TEM (Hitachi H7650) were used to observe the surface morphologies of the fibers. DSC analyses of the samples were carried out with a NETZSCH DSC200F3 with a temperature scanning cycle of 0–80–0 ℃ (at heating and cooling rates of 10 ℃/min) under N_2. The crystallization properties of the samples were characterized using wide-angle XRD diffraction (Philip PW 1710) and POM (Olympus BX51). The luminescence properties of the as-prepared samples were characterized using Gangdong F3800 and Horiba FL3-2-iHR320 fluorescence spectrophotometers. The Raman spectra of samples were tested by Horiba XploRA Laser Raman Spectroscope.

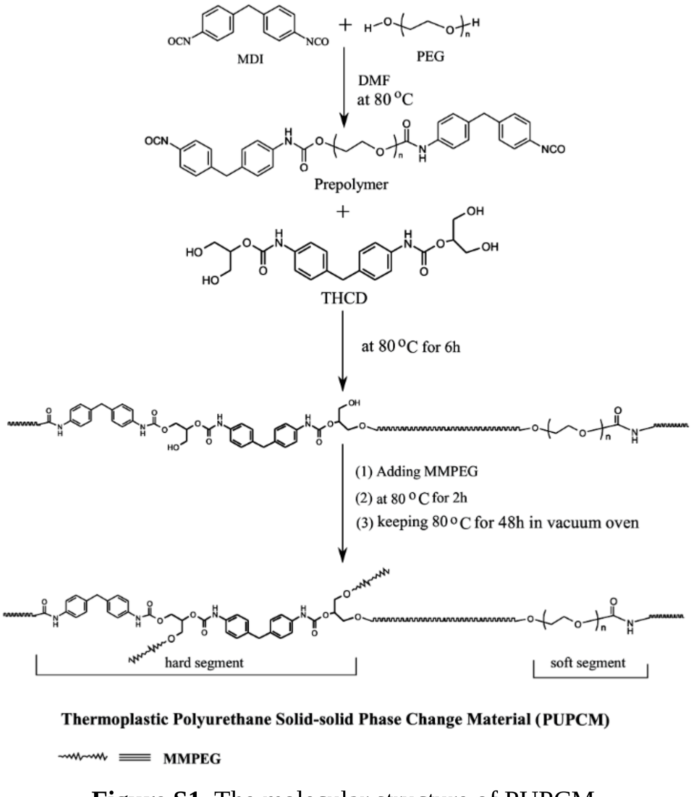

Figure S1. The molecular structure of PUPCM.

Table S1. Crystalline parameters of dual-functional ultrafine composite fibers

Samples	Peak position (2-theta)	FWHM (2-theta)	d(A) (Angstrom)	Area of diffraction peak	Crystallinity (%)
Mixed electrospun ultrafine fiber with 24% solid content	19.135	0.462	4.63	295	20.13
	23.294	1.062	3.82	962	
Mixed electrospun ultrafine fiber with 28% solid content	19.106	0.459	4.64	968	21.82
	23.356	0.905	3.81	306	
Mixed electrospun ultrafine fiber with 32% solid content	18.941	0.707	4.68	716	29.20
	23.121	1.089	3.84	2067	
Parallel electrospun ultrafine fiber with 24% solid content	19.168	0.608	4.63	750	36.32
	23.367	1.134	3.80	2448	
Parallel electrospun ultrafine fiber with 28% solid content	19.086	0.477	4.65	1270	41.84
	23.294	0.834	3.82	2609	
Parallel electrospun ultrafine fiber with 32% solid content	19.154	0.483	4.63	1431	55.52
	23.397	0.822	3.80	2955	

FWHM: full width at half maximum; d (A): lattice plane spacing.

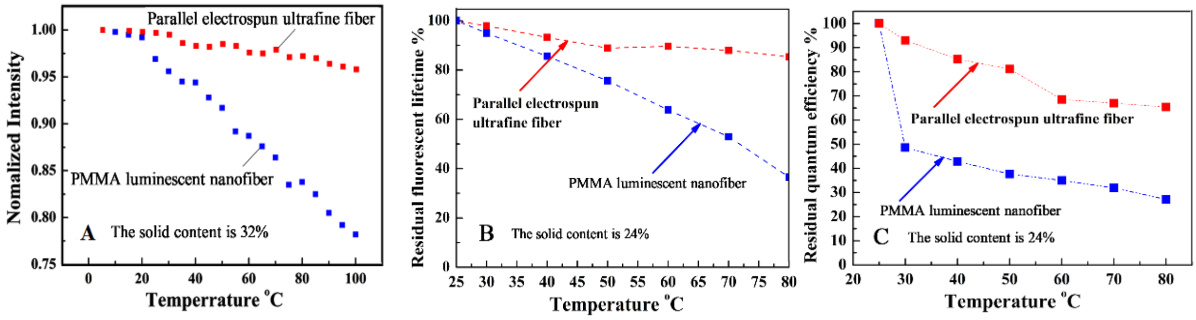

Figure S2. Temperature dependence of luminescence properties for parallel electrospun ultrafine fibers: fluorescence intensity (A), fluorescence lifetime (B), and quantum yield (C).

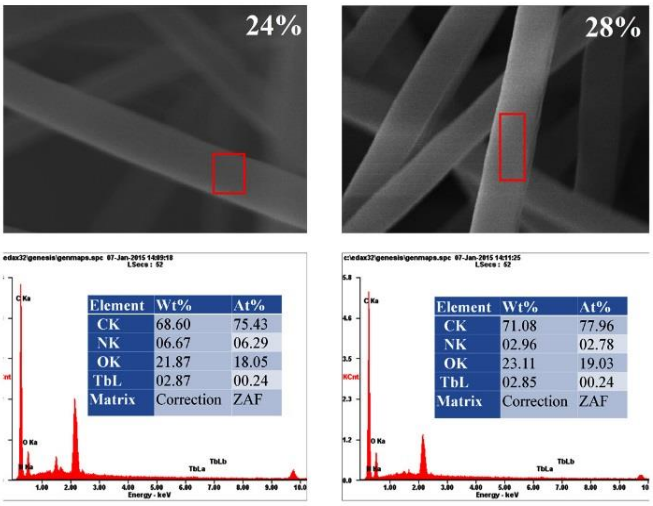

Figure S3. EDS spectra of parallel electrospun dual-functional ultrafine fibers. A: 24% solid content, B: 28% solid content.

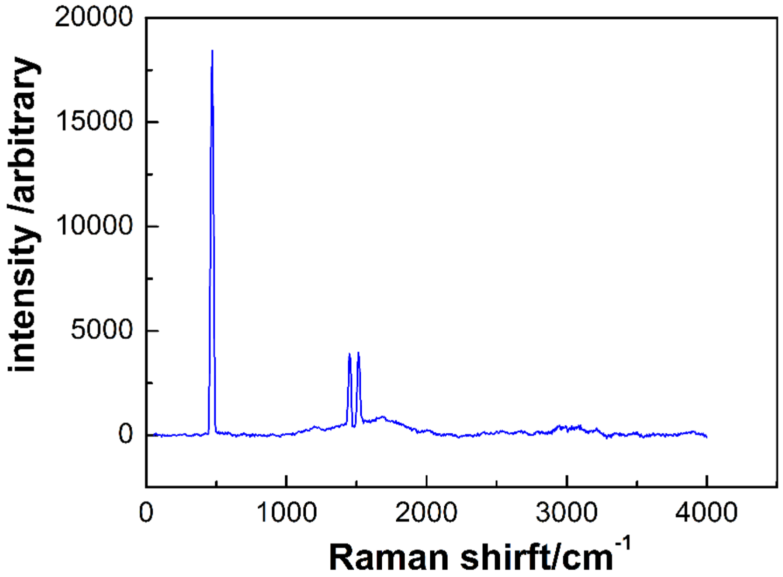

Figure S4. Raman spectrum of parallel electrospun ultrafine fiber with polymer concentrations of 32%.

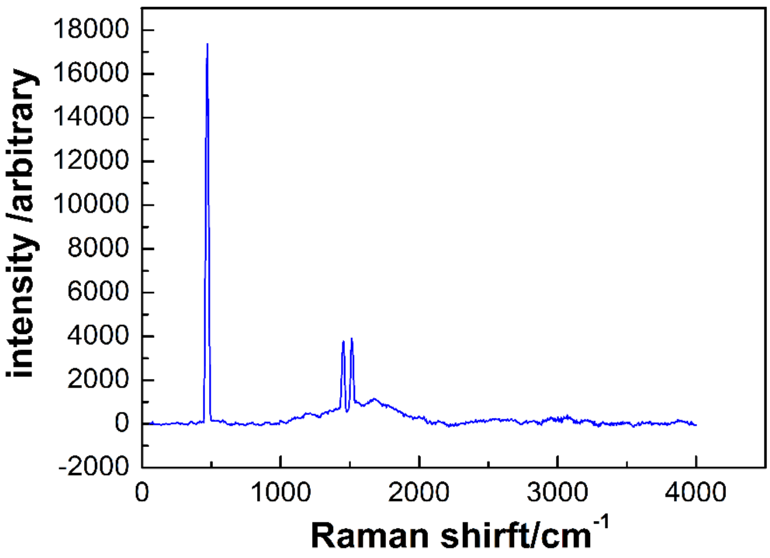

Figure S5. Raman spectrum of mixed electrospun ultrafine fiber with polymer concentrations of 32%.

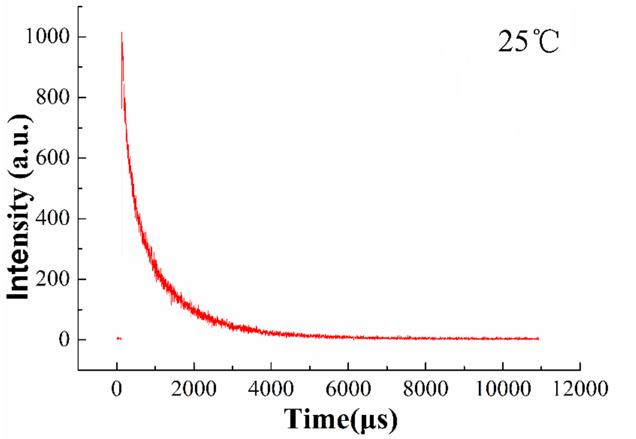

Figure S6. Decay curve of parallel electrospun ultrafine fiber containing 24% solid content at 25 ℃.

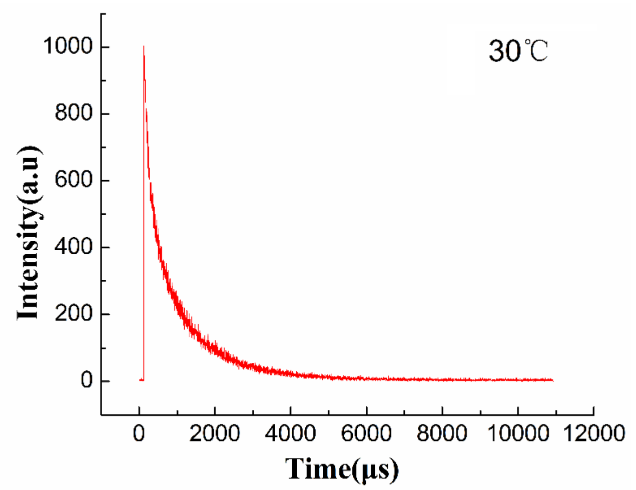

Figure S7. Decay curve of parallel electrospun ultrafine fiber containing 24% solid content at 30 ℃.

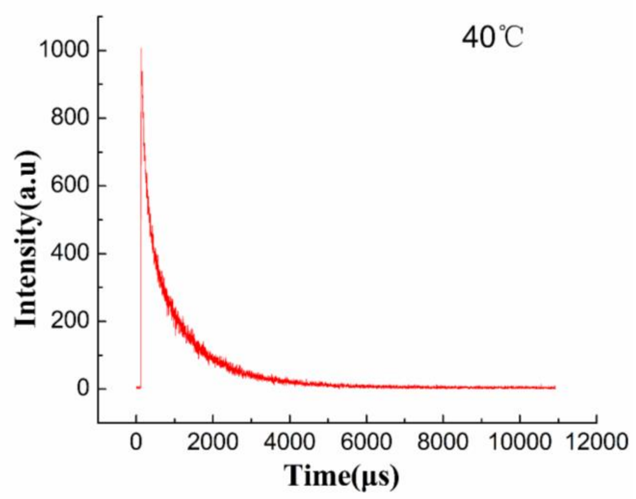

Figure S8. Decay curve of parallel electrospun ultrafine fiber containing 24% solid content at 40 ℃.

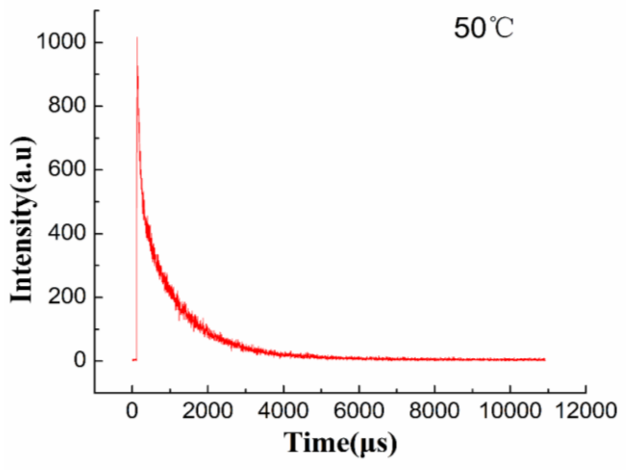

Figure S9. Decay curve of parallel electrospun ultrafine fiber containing 24% solid content at 50 ℃.

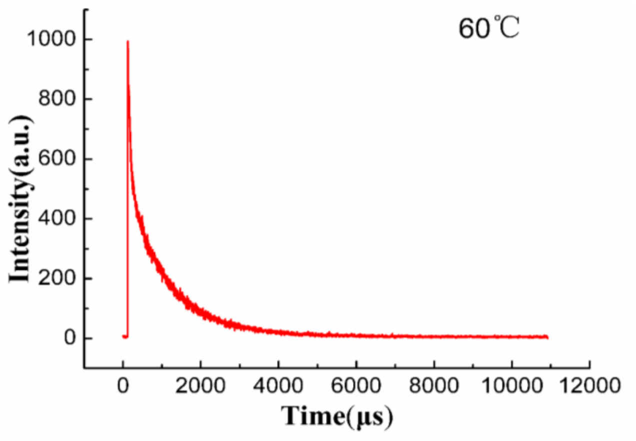

Figure S10. Decay curve of parallel electrospun ultrafine fiber containing 24% solid content at 60 ℃.

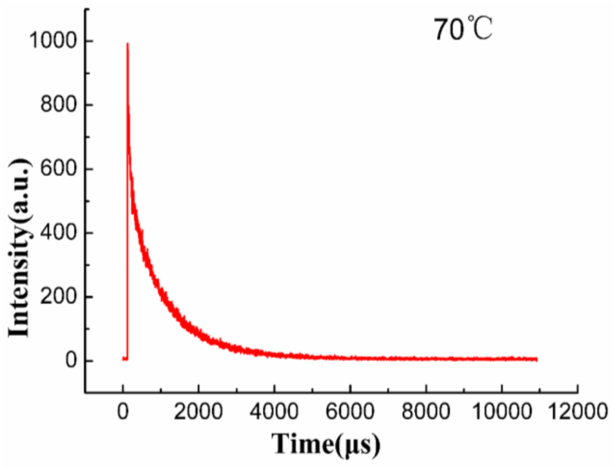

Figure S11. Decay curve of parallel electrospun ultrafine fiber containing 24% solid content at 70 ℃.

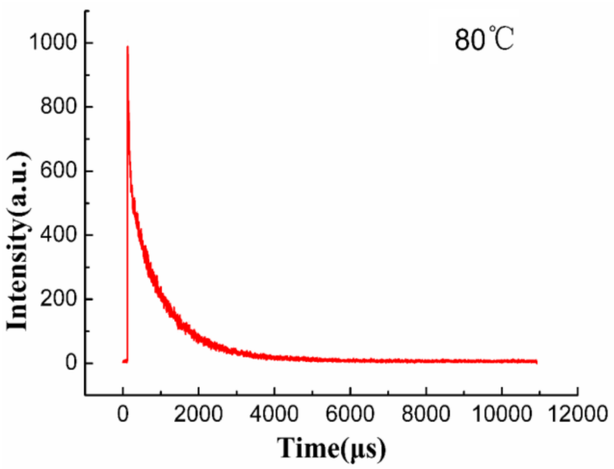

Figure S12. Decay curve of parallel electrospun ultrafine fiber containing 24% solid content at 80 ℃.

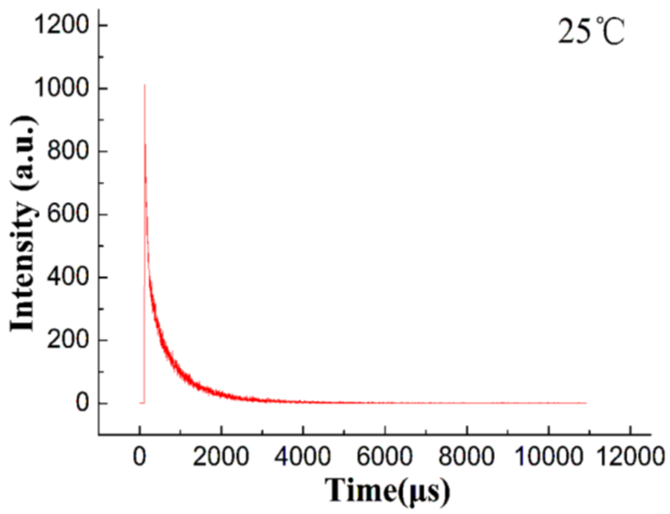

Figure S13. Decay curve of PMMA luminescent nanofiber containing 24% solid content at 25 ℃.

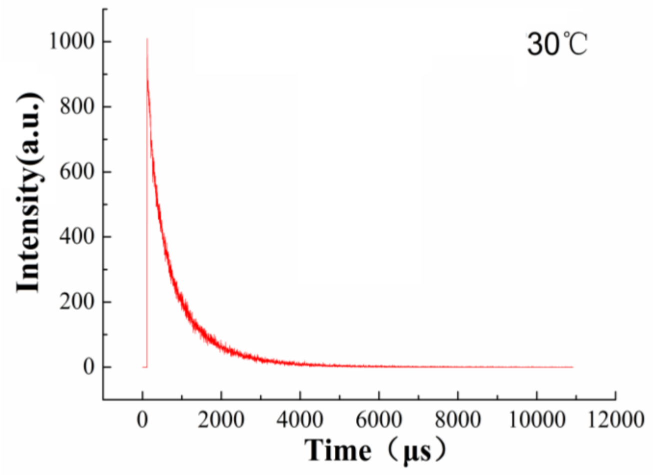

Figure S14. Decay curve of PMMA luminescent nanofiber containing 24% solid content at 30 ℃.

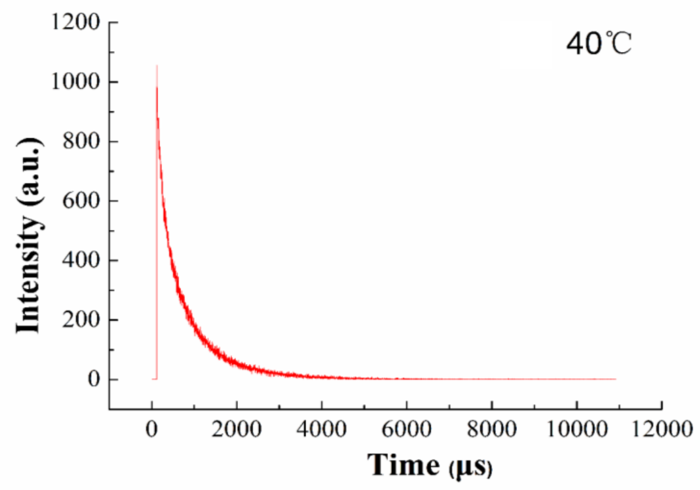

Figure S15. Decay curve of PMMA luminescent nanofiber containing 24% solid content at 40 ℃.

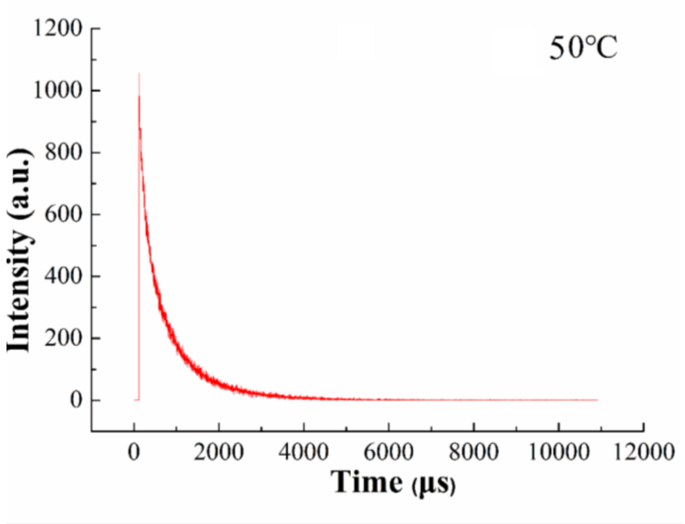

Figure S16. Decay curve of PMMA luminescent nanofiber containing 24% solid content at 50 ℃.

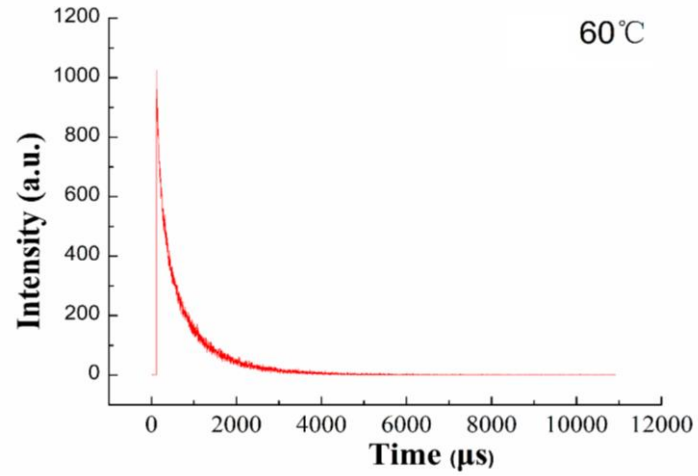

Figure S17. Decay curve of PMMA luminescent nanofiber containing 24% solid content at 60 ℃.

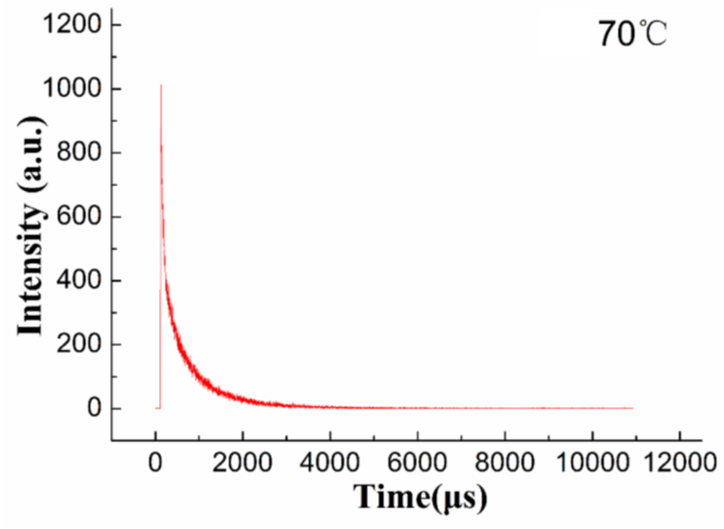

Figure S18. Decay curve of PMMA luminescent nanofiber containing 24% solid content at 70 ℃.

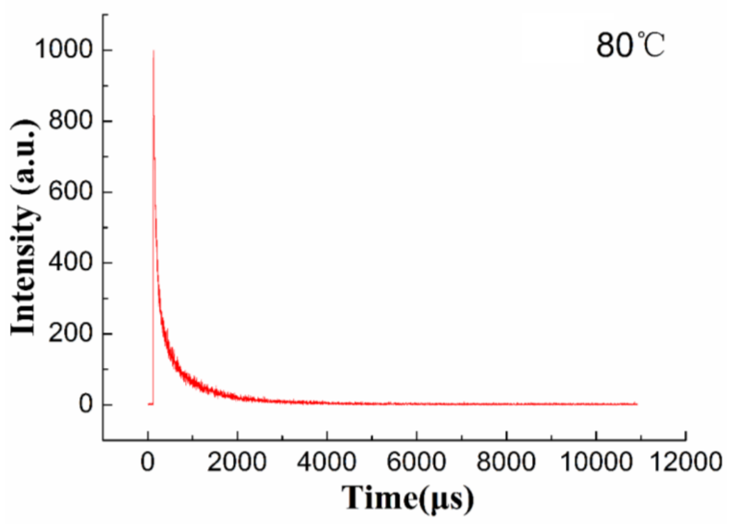

Figure S19. Decay curve of PMMA luminescent nanofiber containing 24% solid content at 80 ℃.
